# Urban morphology dataset of Reunion Island based on the local climate zone classification using satellite imagery and GIS data

**DOI:** 10.1016/j.dib.2025.111747

**Published:** 2025-06-03

**Authors:** Alexandre Lefevre, Bruno Malet-Damour, Fiona Benard, Harry Boyer, Garry Rivière

**Affiliations:** PIMENT Laboratory, University of Reunion Island, 120 Avenue Raymond BARRE, Reunion, Le Tampon 97430, France

**Keywords:** Local climate zone, Urban climate, Small island, Urban planning, Urban heat island, Land cover map

## Abstract

This dataset provides a high-resolution spatial classification of urban morphology on Reunion Island, France, using a 100 m x 100 m grid based on the Local Climate Zone (LCZ) framework. The data were generated through a combination of satellite imagery analysis using the World Urban Database and Access Portal Tools (WUDAPT) method and Geographic Information System (GIS) data integration. The classification was refined using building height and surface fraction data from the BD TOPO 2018 database, improving the accuracy of urban form representation.

The dataset is provided in shapefile format, facilitating its use in GIS software by urban planners, researchers, and policymakers. Each grid point contains geospatial and morphological attributes, including LCZ classification, average building height (m), and built surface fraction (%). These parameters allow researchers to spatially correlate urban morphology with observed temperature data, supporting targeted urban heat island (UHI) studies. The most compact LCZ types (LCZ 1, 2, and 3) are mapped in detail, highlighting zones on the island most exposed to climatic risks. These high-risk areas should be identified and prioritized for climate mitigation strategies.

Beyond UHI analysis, the dataset allows for the identification of land use patterns and urban growth trends by analyzing the spatial distribution of LCZ across the island. Areas of recent expansion, often characterized by transitions from natural zones (LCZ 11, 14) to LCZ 6 or 9 (low-rise and sparsely built, can be geolocated and compared with zoning regulations. This offers urban planners a tool to evaluate whether current development aligns with local urban containment policies and to identify zones at risk of unregulated sprawl.

Because the LCZ classification system is internationally standardized, the dataset also supports comparative urban climate studies. It can be directly used to contrast Reunion’s urban structure with other small island developing states, such as Mauritius, in order to evaluate similarities and differences in urbanization dynamics under similar climatic constraints.

Specifications Table

**Table:** 

Subject	Earth & Environmental Sciences
Specific subject area	*Local Climate Zone Map, Urban Climate, Land Cover Map, Remote sensing, GIS*
Type of data	Raw data - Shapefile at 100 × 100m pixel resolution (.shp)
Data collection	The dataset was created using a combination of remote sensing tools and GIS-based analysis. The initial Local Climate Zone classification was generated using the WUDAPT Level 0 Generator (version from wudapt.org, accessed 2023), which applies a Random Forest classification algorithm to Landsat 8 imagery acquired via Google Earth Engine. A total of 159 manually digitized training areas were delineated using Google Earth Pro based on visual interpretation of land urban morphology, and local field knowledge acquired through site visits. The classification results were then refined using QGIS 3.28 and Python 3.8 scripts. Building footprints and height attributes were extracted from the BD TOPO 2018 database, published by the Institut National de l’Information Géographique et Forestière. Each 100 × 100 m grid cell was attributed morphological metrics based on spatial overlay calculations: mean building height and building surface fraction. Grid cells lacking valid building data or falling outside inhabited zones were excluded from the refinement process.
Data source location	Reunion Island, France is a French overseas territory located in the southwest of the Indian Ocean (bounding box: upper left corner at -20.860994, 55.215189 and lower right corner at -21.3930905, 55.8407771, in EPSG:4326).
Data accessibility	Repository name: Lefevre, Alexandre (2025), “A Local Climate Zone Dataset for urban form analysis over Reunion Island”, Mendeley Data, V2, doi: 10.17632/t4b4mv73gp.2 Data identification number: 10.17632/t4b4mv73gp.2 Direct URL to data: https://data.mendeley.com/datasets/t4b4mv73gp/2
Related research article	[1] Lefevre A, Malet-Damour B, Bénard F, Boyer H, Rivière G. Integrating urban heat island analysis for sustainable urban planning: Insights from Reunion Island. Build Environ 2025;278:112964. 10.1016/j.buildenv.2025.112964.

## 1 Value of the Data

• This dataset enables fine-scale studies of UHI phenomena on Reunion Island, France. It provides a detailed classification of urban morphology through the Local Climate Zones framework, allowing for analysis of temperature differences between rural and urban areas based on morphological parameters. This makes it particularly valuable for understanding climate-related challenges in tropical island contexts.
• By integrating standardized LCZ mapping, the dataset supports comparative studies of UHI effects across tropical and subtropical regions, especially in the Southern Hemisphere. It offers a rare opportunity to investigate the impact of urban overheating on a small island, which remains an underexplored area in urban climate research.
• The dataset facilitates land use analysis and spatial planning assessments. Urban planners and policymakers can use it to evaluate the spatial distribution of built-up areas, assess the alignment of current urban regulations with climate resilience goals, and develop targeted mitigation strategies—such as increasing urban vegetation or limiting high-density developments in heat-prone areas.
• Containing urban sprawl is a critical issue for geographically constrained island territories like Réunion. By combining LCZ classifications with GIS layers (e.g., building height, impervious surfaces), the dataset helps analyze patterns of urban expansion and determine whether cities are developing in a structured or unregulated manner. This insight is crucial for promoting sustainable and climate-adaptive urban growth.
• As LCZ mapping follows a globally recognized methodology, the dataset can be used for benchmarking classification models, validating remote sensing techniques, and refining urban climate models. It serves as a reference for researchers and urban stakeholders interested in improving environmental and thermal resilience in tropical island environments.

## 2 Background

The urban heat island phenomenon is a central topic in urban climate research. It refers to the temperature difference between urban and rural areas, where cities tend to be warmer due to anthropogenic activities and built environment characteristics. This effect has significant implications for urban living conditions, affecting thermal comfort, increasing mortality rates during heat waves, and driving up energy demand for cooling. Understanding and mitigating UHI is thus crucial for sustainable urban planning and land management, particularly in the context of climate change.

Urban climate studies have shown that urban morphology plays a key role in shaping local climate conditions. [2,3] introduced the LCZ classification system, which categorizes urban and natural landscapes into 17 distinct classes. This system has since become a widely adopted framework for UHI studies.

Despite a growing number of LCZ-based studies, small islands such as Réunion Island remain underrepresented in global urban morphology datasets. This limits the capacity to conduct detailed UHI analyses in these settings. The dataset presented here addresses this gap by providing a high-resolution LCZ classification of Réunion Island using satellite imagery and GIS building data. It complements our previously published research article by making the full dataset openly available and reproducible for further urban climate investigations.

## 3 Data Description

The dataset is a shapefile representing a high-resolution map of Réunion Island, structured on a 100 m x 100 m grid. This format allows for easy integration into GIS software, making it accessible for urban planners, researchers, and policymakers. Each grid cell contains multiple attributes that describe the local urban morphology and land use characteristics.

The attributes included in the shapefile are as follows:

• `geometry': The latitude and longitude coordinates of the grid point.
• `LCZ': The *Local Climate Zone* classification of the 100 m x 100 m grid cell, representing urban morphology based on the standardized LCZ framework presented in Fig. 2.
• `Height (m)': The average height (in meters) of buildings within the 100 m x 100 m grid cell.
• ‘BSF (%)': The Buildind Surface Fraction representing the percentage of built-up surface within the 100 m x 100 m grid cell.

**Fig. 1:**
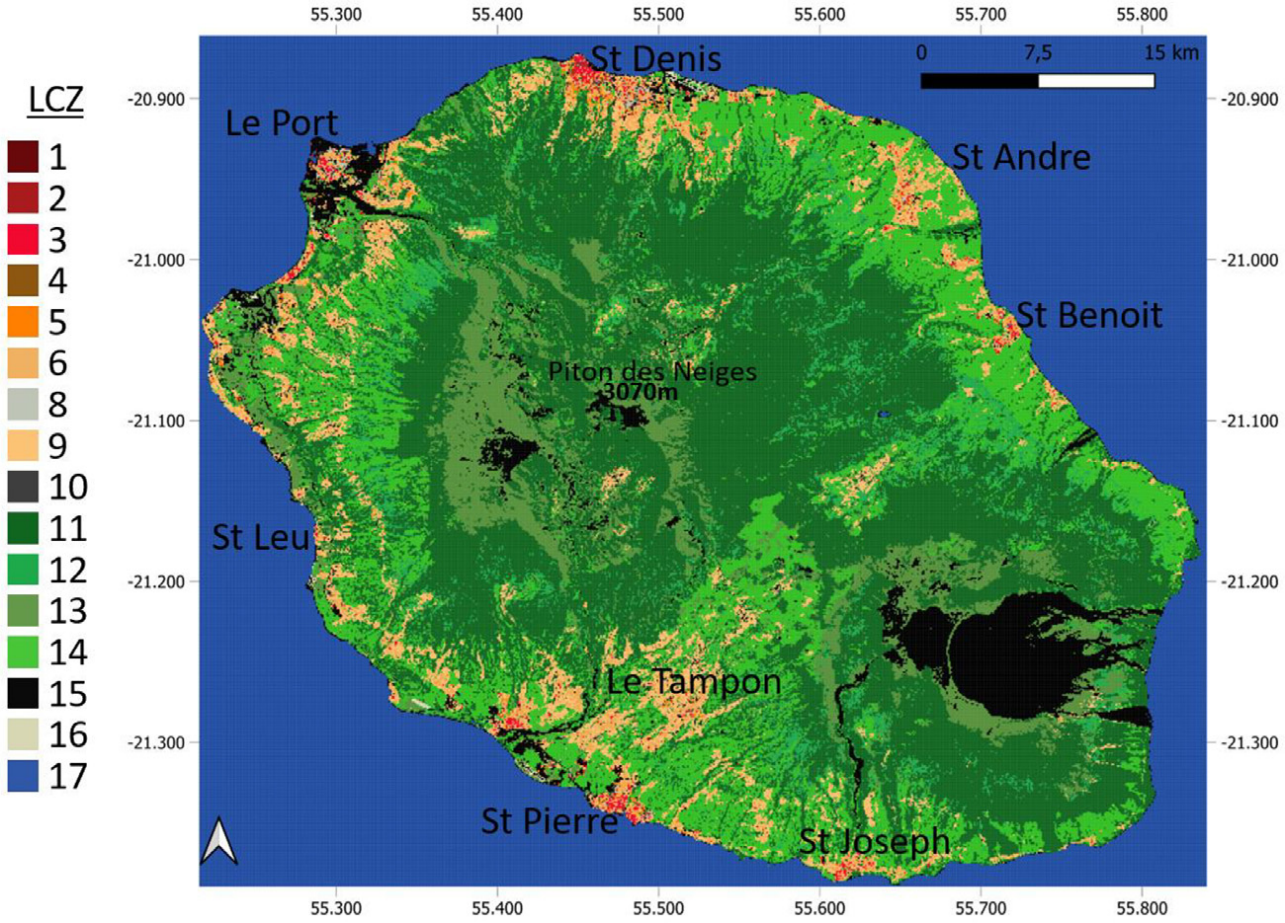
LCZ map of Reunion Island.

**Fig. 2:**
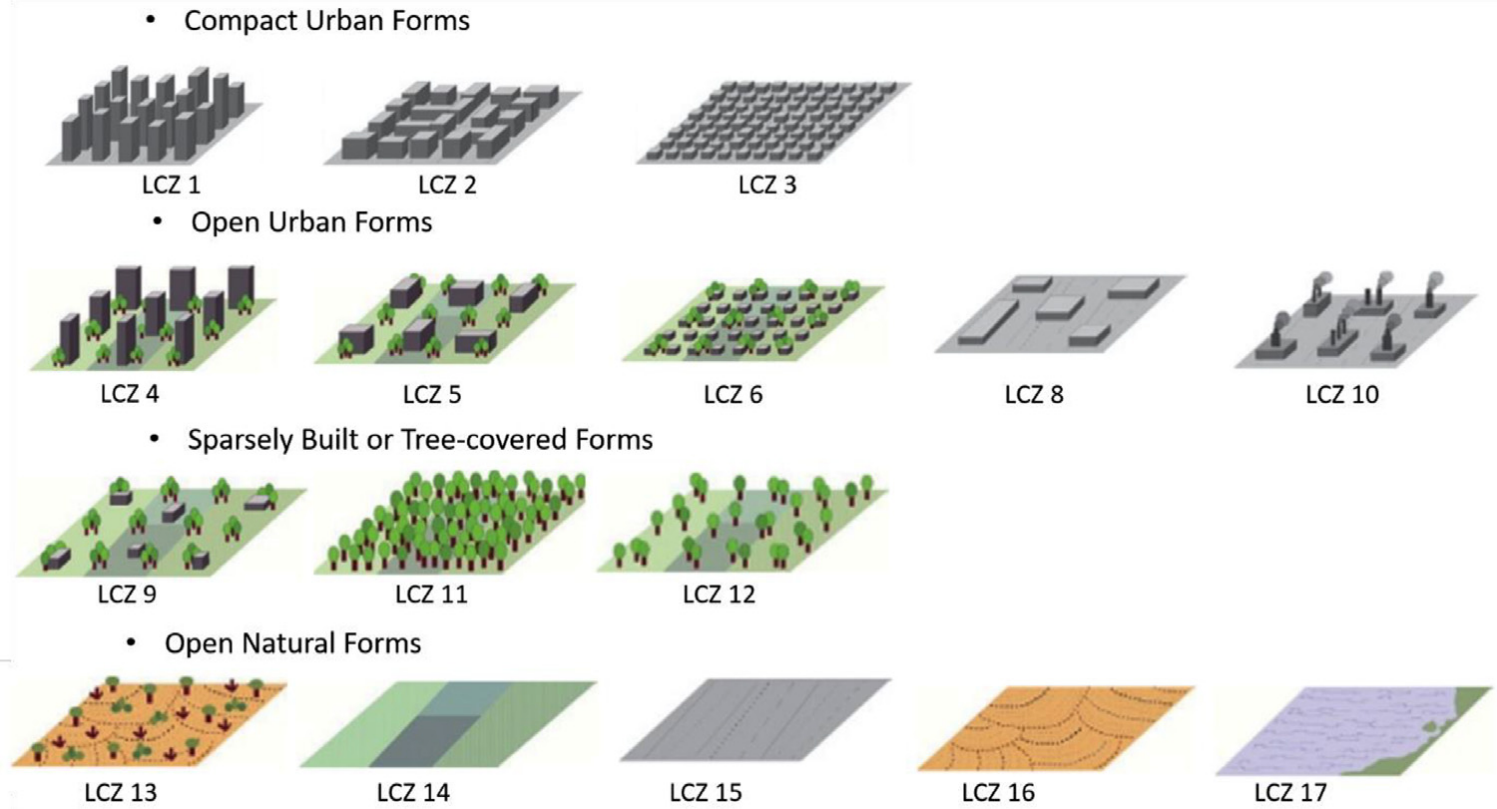
Illustration of the LCZ classes represented in the dataset inspired from [2].

A representation of the LCZ map is given in Fig. 1 to illustrate the shapefile with the different represented classes represented in Fig. 2.

## 4 Experimental Design, Materials and Methods

This section expands, clarifies, and complements the dataset description previously provided in the associated research article [1]. While the original article focused primarily on the scientific analysis of the Urban Heat Island (UHI) phenomenon and its implications for urban planning, this data paper aims to provide a detailed, structured, and reusable presentation of the raw LCZ data.

In particular, it offers a comprehensive description of the methodology used to generate the LCZ classification, which underpins many of the spatial analyses conducted in the main article. This includes step-by-step details of the data sources, preprocessing techniques, classification criteria, validation procedures, and the tools used to assign LCZ classes within the study area.

**Fig. 3:**
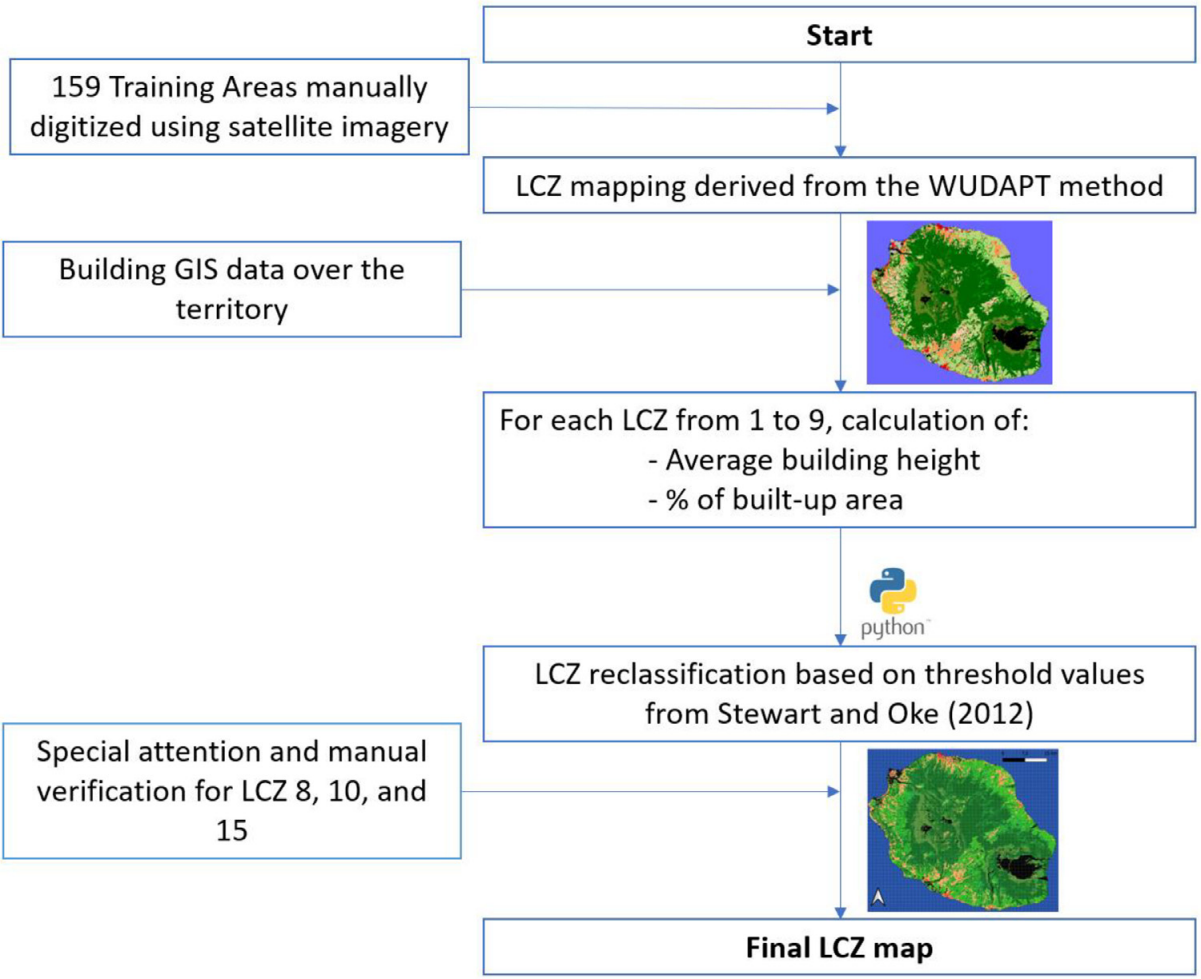
Graphical process of LCZ classification.

The mapping of Local Climate Zones (LCZ) relies on two primary approaches: satellite imagery analysis and GIS data processing as illustrated in Fig. 3. The first method is based on remote sensing technologies, with the World Urban Database and Access Portal Tools (WUDAPT) being the most commonly used framework, developed by [4,5]. WUDAPT utilizes a machine-learning classification algorithm applied to Google Earth© images, leveraging manually defined training areas to generate LCZ maps. The second approach directly classifies LCZs using GIS data, such as building footprints, heights, and land cover attributes. Combining both methods can improve classification accuracy by integrating remote sensing data with in-situ measurements, thereby providing a standardized methodology for defining urban and rural zones.

1. WUDAPT Classification

The initial LCZ classification for Réunion Island was conducted using the WUDAPT framework. Following the methodology described by [5], a total of 159 training areas were selected across the island. These areas were identified and classified based on Google Earth© imagery [6], supplemented by local knowledge and field visits. Each training area was assigned to a specific LCZ category, ensuring comprehensive spatial coverage. All training areas used to generate the LCZ map are openly available through the WUDAPT platform and can be accessed in the corresponding dataset [7].

To train the classification model, between 5 and 20 training areas were provided for each LCZ type present on the island—in line with WUDAPT recommendations to use at least 5 to 15 polygons per class, depending on class variability and seasonal differences in appearance. A three-step iterative classification process was carried out to refine the input zones and optimize accuracy. Following the quality control recommendations, the classification process was repeated three times. Each iteration included revision and addition of training areas to better represent underperforming LCZ types. This allowed for progressive improvement in classification accuracy by increasing the representativity of the training data.

However, LCZ type 7—characterized by informal settlements with lightweight structures—was absent on the island and thus excluded from the classification.

2. Analysis of the WUDAPT Classification

As part of the WUDAPT LCZ Generator process, model training and validation were carried out using a stratified 25-fold bootstrap cross-validation approach. For each bootstrap run, 70% of the training area polygons were randomly selected (while preserving class distributions) for training, and the remaining 30% were used for validation. This repetition allowed for the generation of confidence intervals around key accuracy metrics, including Overall Accuracy (OA), Urban Accuracy (OAu), Built vs. Natural Accuracy (OAbu), and Weighted Accuracy (OAw).

It is important to note that, while the OA for this classification reached approximately 0.68, this figure only reflects model performance within the training area polygons. Areas outside these polygons are not subject to the same internal validation and may exhibit greater uncertainty. Furthermore, potential errors in training data—such as visual confusion between morphologically similar classes like LCZ 3 and 6—can inflate OA scores and reduce generalization capacity.

The performance of the WUDAPT classification was first assessed using global accuracy metrics, which provide an overview of the model's effectiveness at various levels of detail.

• The Overall Accuracy (OA) reached approximately 0.68, indicating that about 68% of all samples were correctly classified.
• When focusing specifically on urban LCZ types, the Urban-Class Accuracy (OAu) dropped slightly to 0.65, highlighting the model’s relative difficulty in distinguishing between urban forms.
• However, the Urban vs. Natural Classification Accuracy (OAbu) was much higher, around 0.90, showing that the model is very effective at separating built-up from non-built-up areas.
• The Weighted Accuracy (OAw), which compensates for class imbalance, reached 0.92, reinforcing the overall robustness of the classification despite challenges with specific LCZ types.

**Fig. 4:**
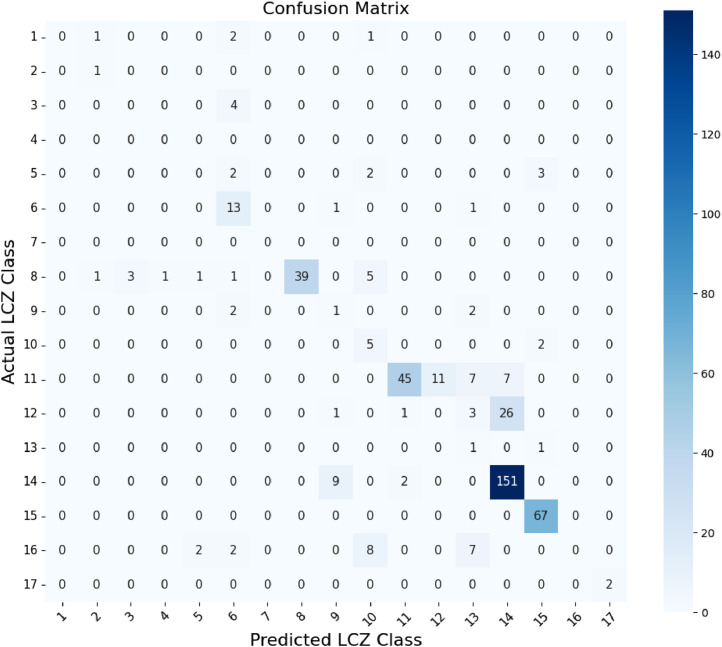
WUDAPT classifier accuracy for each LCZ.

**Fig. 5:**
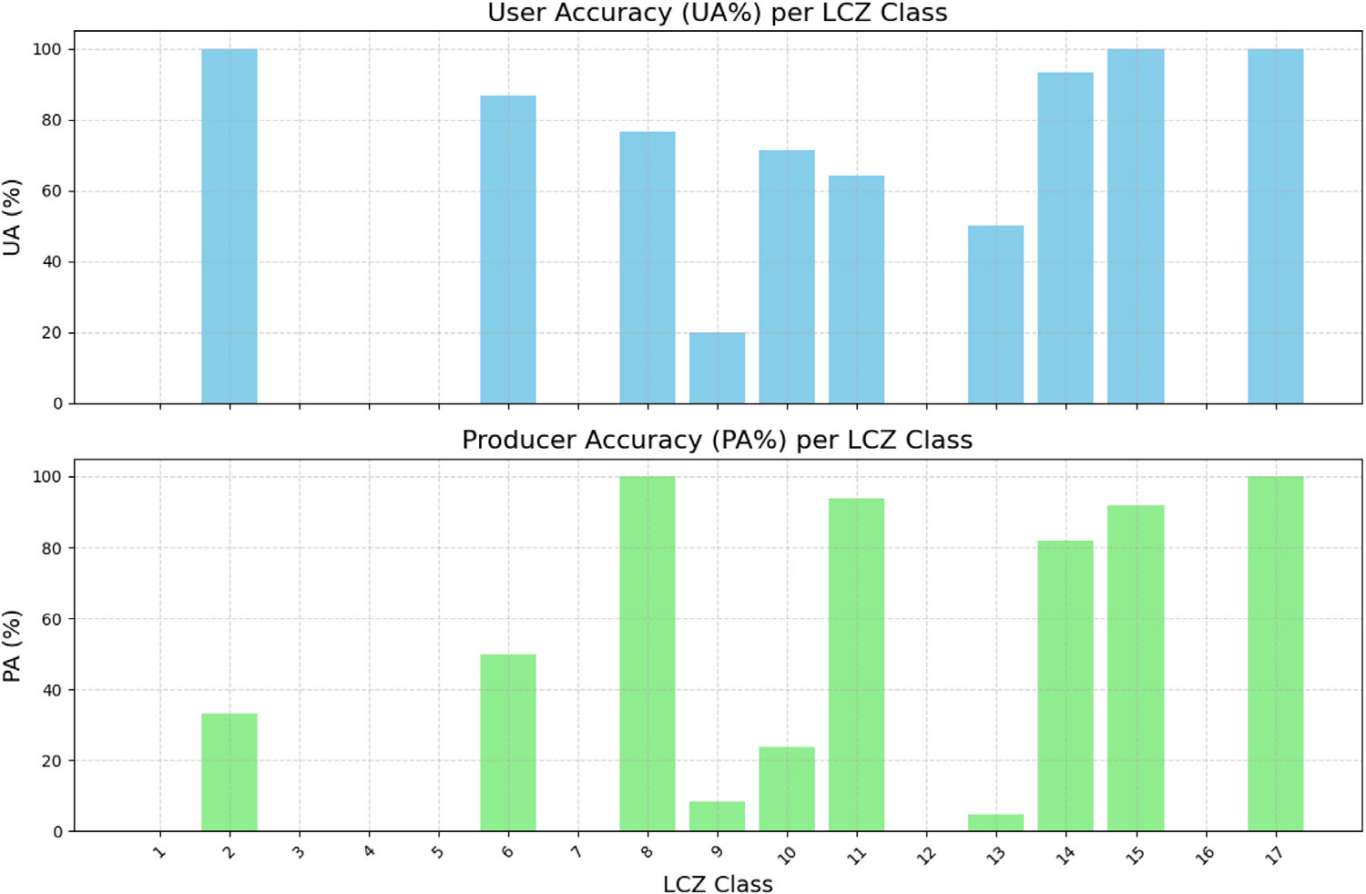
User and producer accuracy of the WUDAPT classification.

To complement these aggregate indicators, we used two visualizations: a confusion matrix (Fig. 4) and a bar chart of User’s and Producer’s Accuracy (UA and PA) (Fig. 5). These allow for a more detailed class-by-class performance analysis. These two figures provide a comprehensive understanding of the classification's strengths and limitations across the 17 LCZ types.

The confusion matrix (Fig. 4) presents the raw distribution of correct and incorrect classifications by comparing predicted LCZ classes (columns) against reference classes (rows). The diagonal elements represent correct predictions; off-diagonal elements indicate confusion between classes. This visualization highlights the structure of classification errors, particularly how frequently one class is mistaken for another.

From this matrix, we observe that urban classes LCZ 1, 3, 5, 8 and 9 are poorly recognized, with most of their samples misclassified as LCZ 6 (Open Low-Rise) or LCZ 10 (Heavy Industry). For instance:

• LCZ 3 (Compact Low-Rise) was entirely misclassified as LCZ 6.
• LCZ 5 (Open Mid-Rise) saw dispersion across LCZ 6 and LCZ 10.
• LCZ 9 (Sparsely Built) was confused with both LCZ 6 and LCZ 13.

These misclassifications reflect the spectral and morphological similarities among certain urban typologies when viewed from medium-resolution satellite imagery. WUDAPT tends to overgeneralize compact and mid-rise zones into broader open low-rise categories, likely due to limitations in spatial resolution and contextual data.

In Fig. 4, the UA and PA metrics provide a quantitative summary of these observations:

• User Accuracy (UA) measures the reliability of the predicted class (i.e., how often a class label is correct).
• Producer Accuracy (PA) measures the completeness of the classification for a given class (i.e., how often real instances are correctly labeled).

Here, we see stark disparities:

• LCZ 2, 15, and 17 reached perfect UA and PA (100%), indicating highly accurate and unambiguous classification for these classes.
• In contrast, LCZ 3, 4, 5, 12, and 16 had extremely low UA and PA, with several showing 0% for one or both metrics.

For example:

• LCZ 3 had UA and PA of 0%, indicating total failure to detect or correctly predict this class.
• LCZ 12 (Scattered Trees) was particularly problematic: it was largely misclassified as LCZ 14 (Low Vegetation) and LCZ 13 (Bush/Scrub), yielding 0% UA and PA.
• LCZ 9 and LCZ 5 showed UA values of 20% and 0%, respectively, again reinforcing the confusion among low-density built-up types.

These findings underscore a broader issue: WUDAPT excels at distinguishing natural versus built-up zones but fails to reliably separate urban subtypes, especially in tropical environments like Réunion Island, where vegetation is abundant and spatial patterns are complex. The spectral resemblance between forested, vegetated, and mixed-use zones makes fine-grained classification challenging with limited input features.

In contrast, classes with more distinct spectral profiles and consistent spatial patterns—such as LCZ 14 (Low Vegetation, UA = 93.2%), LCZ 15 (Bare Soil/Rock, UA = 100%), and LCZ 6 (Open Low-Rise, UA = 86.7%)—were much better classified.

Together, Fig. 4, Fig. 5 reveal that while WUDAPT provides a broadly effective urban-natural classification, it struggles with intra-urban variability and natural class nuances.

3. GIS classification

To address these limitations and enhance classification accuracy— particularly for urban LCZ types —GIS data from BD TOPO 2018, an open-access database provided by the Institut National de l’Information Géographique (IGN France), was integrated into the workflow. This dataset offers detailed vector information on building heights and footprints across Réunion Island. To incorporate this GIS information, we used a combination of Python scripts and QGIS software, enabling the integration of the BD TOPO data with the LCZ raster layer generated through the WUDAPT methodology. This hybrid approach allowed for the refinement of urban class boundaries and supported a more accurate interpretation of urban morphology patterns.

For each 100 m x 100 m grid cell classified as an urban LCZ using WUDAPT, two key indicators were computed using Python scripts in QGIS:

1. The average Building Height within the grid cell (in meters),
2. The Building Surface Fraction (percentage of land occupied by buildings)

These indicators were then used to refine LCZ classification based on criteria established by [3], as summarized in Table 1.

**Table 1:** LCZ classification and morphological characteristics.

Local Climate Zone	Building Surface Fraction (%)	Building Height (m)
LCZ 1 – Compact high-rise	40–60	> 25
LCZ 2 – Compact mid-rise	40–70	8–20
LCZ 3 – Compact low-rise	40–70	3–8
LCZ 4 – Open high-rise	30–40	> 25
LCZ 5 – Open mid-rise	20–40	8–20
LCZ 6 – Open low-rise	20–40	3–8
LCZ 8 – Large low-rise	< 10	3–10
LCZ 9 – Sparsely built	10–20	3–8

**Fig. 6:**
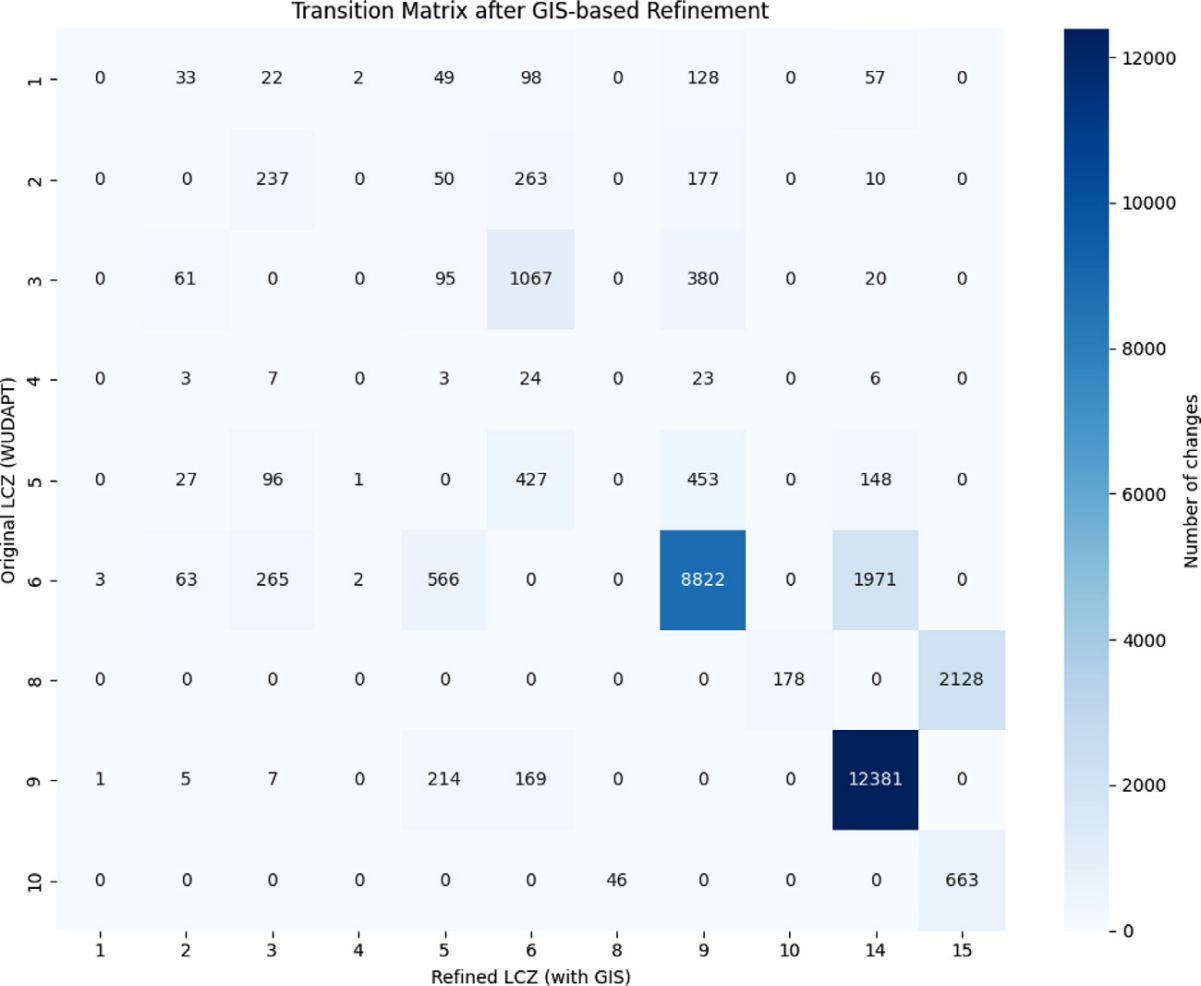
Matrix of transition after GIS refinement.

The refinement process led to a substantial reclassification of the built-up areas: approximately 60% of the grid cells initially labeled with an urban LCZ type were reassigned to a different class based on the GIS-derived indicators. The transition matrix between the initial and refined classifications is presented in Fig. 6.

The most significant change observed was a massive reclassification from LCZ 9 (Sparsely built) to LCZ 14 (Low plants), with 12,381 occurrences, reflecting the correction of false positives caused by features such as greenhouses, fences, or isolated rural constructions misidentified as built-up zones. Another prominent transition involved LCZ 6 (Open mid-rise) being reassigned to LCZ 9 (Sparsely built) in 8,822 cases, illustrating the impact of refined surface fraction thresholds.

Other notable changes included:

• LCZ 8 (Large low-rise) to LCZ 15 (Bare soil) (2,128 cases),
• LCZ 6 to LCZ 14 (1,971 cases),
• LCZ 3 (Compact low-rise) to LCZ 6 (1,067 cases),
• LCZ 10 (Heavy industry) to LCZ 15 (663 cases).

These transitions highlight the model’s sensitivity to building density and height metrics, particularly in zones where visual interpretation from satellite imagery is ambiguous. Smaller but recurrent adjustments also occurred between LCZ types with similar urban typologies, such as transitions between LCZ 3, 5, and 6.

This refinement confirms the value of integrating detailed GIS datasets to correct remote sensing-derived misclassifications and better align LCZ maps with actual urban morphology.

4. Final Assessment and Focus on LCZ 10

Due to a lack of distinguishing features for LCZ 10—typically associated with heavy industrial areas—and the absence of specific data on anthropogenic CO₂ emissions necessary for its accurate identification, this class was examined separately and manually. Only heavy industrial facilities such as the sugar factories of Le Gol (approximately at -21.279569,55.397582) and Bois Rouge (approximately at -20.914139,55.635776), as well as selected pixels within industrial zones in Le Port (around -20.933344,55.290725), were retained under LCZ 10.

All other points initially classified as LCZ 10 were carefully reviewed using satellite imagery and reassigned to other LCZ classes based on the criteria established in Table 1. These adjustments ensure a more consistent and realistic representation of the built environment.

The final LCZ map, incorporating both WUDAPT-based classification and GIS-derived refinements, was generated at a 100-meter spatial resolution (Fig. 1). This high-resolution product provides a detailed and reliable representation of urban morphology across Réunion Island, offering a valuable dataset for future studies in urban climatology [8], land-use and spatial planning [9], urban development [1], and environmental management.

## Limitations

One primary limitation of the dataset lies in its spatial resolution of 100 × 100 m. Each grid cell is assigned a single, homogeneous LCZ class, as defined in [2]. However, urban environments—especially in complex and organically grown settings like Réunion Island—are rarely homogeneous at this scale. Unlike many Western European cities, which tend to follow structured and hierarchical urban planning models, urban development on Réunion often occurs in a more fragmented or linear pattern along road networks. As a result, a single grid cell may encompass a mixture of built forms, land uses, or building heights, reducing the precision of the classification. Similar challenges have been observed in other tropical cities such as Colombo [10] and Nagpur [11], where hybrid or transitional urban typologies are common. Future efforts could benefit from a finer classification scale—such as delineation at the building block level as in [12]—or the introduction of hybrid or intermediate LCZ classes better suited to such complex urban fabrics.

Another limitation is the temporal mismatch between the datasets used. The LCZ classification was based on satellite imagery from 2022, while the GIS building data used for refinement came from the 2018 BD TOPO database. This four-year gap may result in the omission of recent urban expansions, new constructions, or demolitions, particularly in areas experiencing rapid growth or informal development. Consequently, the current dataset might not fully reflect the most recent state of the urban landscape. Future updates of the map should aim to integrate more temporally aligned datasets, ideally incorporating continuously updated GIS information and high-resolution Earth observation data.

Despite these limitations, the resulting LCZ map remains a valuable resource. It provides the first harmonized and high-resolution representation of urban morphology across Réunion Island. This map serves as a foundational dataset for various applications, including urban heat island analysis, climate resilience planning, and environmental management. It also sets the stage for future enhancements, such as dynamic updates, increased spatial detail, and integration of climate-relevant indicators. As such, it represents an essential step toward improved understanding and sustainable planning in tropical island environments.

## Ethics Statement

The authors have read and follow the ethical requirements for publication in Data in Brief and confirming that the current work does not involve human subjects, animal experiments, or any data collected from social media platforms.

## CRediT authorship contribution statement

**Alexandre Lefevre:** Conceptualization, Methodology, Software, Validation, Investigation, Investigation, Resources, Data curation, Writing – original draft, Writing – review & editing. **Bruno Malet-Damour:** Writing – review & editing, Funding acquisition. **Fiona Benard:** Methodology, Investigation, Resources, Writing – review & editing. **Harry Boyer:** Writing – review & editing. **Garry Rivière:** Writing – review & editing, Supervision, Project administration, Funding acquisition.

## Data Availability

Mendeley DataA Local Climate Zone Dataset for urban form analysis over Reunion Island (Original data).

## References

[1] Lefevre A., Malet-Damour B., Bénard F., Boyer H., Rivière G. (2025). Integrating urban heat island analysis for sustainable urban planning: insights from reunion Island. Build. Environ..

[2] Stewart I.D., Oke T.R. (2012). Local climate zones for urban temperature studies. Bull. Am. Meteorol. Soc..

[3] Stewart I.D., Oke T.R., Krayenhoff E.S. (2014). Evaluation of the ‘local climate zone’ scheme using temperature observations and model simulations: evaluation of the ‘local climate zone’ scheme. Int. J. Climatol..

[4] Bechtel B., Alexander P., Böhner J., Ching J., Conrad O., Feddema J. (2015). Mapping local climate zones for a worldwide database of the form and function of cities. ISPRS Int. J. Geo Inf..

[5] Demuzere M., Kittner J., Bechtel B. (2021). LCZ generator: a web application to create local climate zone maps. Front. Environ. Sci..

[6] D. Wuthrich, Google Earth Pro. | EBSCOhost 2006;16:30. https://openurl.ebsco.com/contentitem/gcd:19906899?sid=ebsco:plink:crawler&id=ebsco:gcd:19906899 (accessed May 21, 2025).

[7] A. Lefevre, WUDAPT Level 0 training data for Tampon (Reunion), submitted to the LCZ generator. 2024. This dataset is licensed under CC BY-NC-SA, and more information is available at https://lcz-generator.rub.de/factsheets/e5b4ee42b717f505d98c2371c7cd969f088b19b6/e5b4ee42b717f505d98c2371c7cd969f088b19b6_factsheet.html.

[8] Lefevre A., Malet-Damour B., Boyer H., Rivière G. (2024). Advancing urban microclimate monitoring: the development of an environmental data measurement station using a low-tech approach. Sustainability.

[9] Dupuy S., Gaetano R., Le Mézo L. (2020). Mapping land cover on Reunion Island in 2017 using satellite imagery and geospatial ground data. Data Br..

[10] Perera N.G.R., Emmanuel R.A (2018). Local climate zone” based approach to urban planning in Colombo, Sri Lanka. Urban Clim..

[11] Kotharkar R., Bagade A. (2018). Local climate zone classification for Indian cities: a case study of Nagpur. Urban Clim..

[12] Bocher E., Bernard J., Wiederhold E., Leconte F., Petit G., Palominos S. (2021). GeoClimate: a geospatial processing toolbox for environmental and climate studies. J. Open Source Softw..

